# Virulence and Antimicrobial-Resistant Gene Profiles of *Salmonella* spp. Isolates from Chicken Carcasses Markets in Ibague City, Colombia

**DOI:** 10.1155/2024/4674138

**Published:** 2024-08-23

**Authors:** Kelly Johanna Lozano-Villegas, Iang Schroniltgen Rondón-Barragán

**Affiliations:** ^1^ Immunobiology and Pathogenesis Research Group Faculty of Veterinary Medicine and Zootechnics University of Tolima, Altos the Santa Helena, A.A 546, Ibagué 730006299, Tolima, Colombia; ^2^ Poultry Research Group Laboratory of Immunology and Molecular Biology Faculty of Veterinary Medicine and Zootechnics Universidad del Tolima, Santa Helena Highs, Ibagué 730006299, Tolima, Colombia

## Abstract

*Salmonella* spp. is one of the leading causes of foodborne bacterial infections, with major impacts on public health and healthcare system. *Salmonella* is commonly transmitted via the fecal-to-oral route, and food contaminated with the bacteria (e.g., poultry products) is considered a common source of infection, being a potential risk for public health. The study aims to characterize the antimicrobial resistance- and virulence-associated genes in *Salmonella* isolates recovered from chicken marketed carcasses (*n* = 20). The presence of 14 antimicrobial and 23 virulence genes was evaluated using end-point PCR. The antimicrobial genes were detected in the following proportion among the isolates: *bla*_TEM_ 100%, *dfrA1* and *bla*_CMY2_ 90% (*n* = 18), *aadA1* 75% (*n* = 15), *sul1* and *sul2* 50% (*n* = 10), *floR* 45% (*n* = 9), *qnrD* 20% (*n* = 4), and *aadA2* 15% (*n* = 3). *catA*, *sul3*, *qnrS*, and *aac(6′)-Ib* genes were absent in all isolates. Regarding virulence-associated genes, all *Salmonella* strains contain *invA*, *fimA*, *avrA*, *msgA*, *sopB*, and *sopE*. The *cdtB* gene was present in 95% (*n* = 19) of isolates, whereas *spvC* and *spvB* were present in 55% (*n* = 11). Other virulence genes such as *spiC*, *lpfC*, *lpfA*, and *csgA* were present in 90% (*n* = 18) of strains. The presence of antimicrobial and virulence genes in several *Salmonella* strains in chicken meat suggests the potential pathogenicity of the strains, which is relevant given the possibility of cross-contamination which represents a significant threat to public health.

## 1. Introduction

Foodborne diseases caused by microorganisms are a significant global public health concern [1]. According to the Centers for Disease Control and Prevention (CDC), foodborne pathogens with the highest annual burden of disease and overall impact on public health are *Campylobacter*, *Listeria monocytogenes*, *Salmonella*, *STEC*, *Shigella*, *Vibrio*, and *Yersinia* [2, 3]. *Salmonella* spp. infections are among the leading causes of foodborne bacterial infections, with major impacts on human health and the economy [4–6]. *Salmonella* remains one of the most burdensome foodborne pathogens globally, with an estimated 200 million to over 1 billion infections, 93 million gastroenteritis cases, and 155,000 fatalities [7].

Currently, more than 2600 different serotypes of *Salmonella* have been identified, but only a small number of these are commonly associated with foodborne illnesses in humans [8]. In clinical human medicine*, Salmonella* serotypes can be grouped into typhoidal serotypes (*Salmonella* Typhi and *Salmonella* Paratyphi A, B, C) and non-typhoidal *Salmonella* serotypes (NTS) (caused by other *Salmonella* strains) [9, 10]. The typhoidal *Salmonella*s are invasive and cause systematic infection [11, 12]. In contrast, NTS serotype infections cause diarrhea, nausea, vomiting, abdominal pain, myalgia, and arthralgia and are usually self-limiting, although immunocompromised patients can develop a sepsis [13, 14].

The route of *Salmonella* spp. transmission is commonly through a fecal-to-oral route with the consumption of contaminated food or water [15, 16]. Many foods containing *Salmonella* are derived from animals (such as eggs, poultry, seafood, and meat), and the transfer of bacteria to these products occurs as a result of cross-contamination during food processing [17, 18]. Epidemiological studies have identified poultry meat as the main vehicle for *Salmonella* infection [17, 19]. Poultry meat serves as a good environment for the growth of pathogenic *Salmonella* spp. due to its high nutrient content, pH of 5.5 to 6.5, and water [7, 20]. However, the prevalence of *Salmonella* in poultry products depends on the type of poultry store (supermarkets and fresh food markets) [21].

Currently, several studies have identified that *Salmonella* isolates from chicken farms carry mobile genetic elements (MGEs) that contribute to increased pathogenicity and antimicrobial resistance [22–25]. MGEs include plasmids, integrons, and transposons, which play a crucial role in the dissemination of antibiotic resistance genes (ARGs) and the development of biofilm-mediated antibiotic resistance [26]. *Salmonella* pathogenicity is mediated by virulence genes, which facilitate the survival, colonization, and damage of the host [27]. Consequently, identification and assessment of pathogenic potential and antimicrobial resistance of *Salmonella* serovars in chicken and poultry products is crucial for disease assessment and epidemiological surveillance [21]. Thus, this study aimed to characterize the antimicrobial resistance- and virulence-associated genes in *Salmonella* isolates recovered from chicken carcasses marketed in Ibague, Colombia.

In Colombia, a study on 1.003 retail broiler chicken carcasses reported that 27% were contaminated with *Salmonella*, indicating a risk of infection from poultry products in the country [28]. However, the prevalence of *Salmonella* spp. in chicken carcasses varied among Colombian cities, ranging from 0% to 57% [28]. For example, in Ibague (central region of Colombia), *Salmonella* Enteritidis was identified as a significant source of salmonellosis, particularly linked to contaminated eggs and raw chicken meat, emphasizing the genetic relationship between isolates from poultry and human gastroenteritis cases [29]. Additionally, studies on raw chicken meat sold in Ibague revealed a high prevalence of *Salmonella*, with specific serotypes such as *S.* Paratyphi B, *S.* Hvittingfoss, and *S.* Muenster being predominant and posing a potential threat to human health due to multidrug resistance patterns [30]. Thus, this study aimed to characterize the antimicrobial resistance- and virulence-associated genes in *Salmonella* isolates recovered from chicken carcasses marketed in Ibague, Colombia.

## 2. Materials and Methods

### 2.1. Ethical Approval

Ethical approval was not required for this study. *Salmonella* spp. strains were obtained from the Bacterial Strain Collection at the Laboratory of Immunology and Molecular Biology (Universidad del Tolima).

### 2.2. *Salmonella* Strains

Twenty strains of *Salmonella* previously isolated from broiler carcasses marketed at Ibague, Colombia, were used in this study [31]. The strains used were previously serotyped using the Kauffmann–White scheme as *Salmonella* Paratyphi B (*n* = 5), *Salmonella* Manhattan (*n* = 1), *Salmonella* Bovismorbificans (*n* = 1), *Salmonella* Typhimurium (*n* = 2), *Salmonella* Othmarschen (*n* = 1), *Salmonella* Newport (*n* = 1), *Salmonella* Hvittingfoss (*n* = 5), *Salmonella* Heidelberg (*n* = 1), and *Salmonella* Muenster B (*n* = 3).

### 2.3. Genomic DNA Extraction and Molecular Confirmation of *Salmonella*

Genomic DNA (gDNA) was extracted from fresh bacterial colonies using Wizard® Genomic DNA Purification Kit (Promega, USA) according to the manufacturer's conditions. DNA samples were stored at −20°C prior to use. Molecular confirmation of *Salmonella* isolates was performed by amplification of a 284 bp fragment of the *invA* gene (accession number: M90846.1) by endpoint polymerase chain reaction (PCR), using *Salmonella Enteritidis* (ATCC 13076®) as positive control and *E. coli* (ATCC 25922®) as negative control [31].

### 2.4. Molecular Detection of Antibiotic Resistance Genes and Virulence-Associated Genes

Fourteen antibiotic resistance genes (ARGs) were examined in all isolates by endpoint PCR using specific primers described in Table 1. The ProFlex™ PCR System (Applied Biosystems, Waltham, MA, USA) was utilized for conducting the reactions, using a final volume of 25 *μ*L made up of 14,875 *µ*L deionized distilled water, 5 *μ*L of Flexi Buffer 5× Colorless GoTaq® (Promega, Madison, WI, USA), 1 *µ*L of dNTPs (Invitrogen, Carlsbad, CA, USA), 1 *µ*L of each primer (forward and reverse) (10 pmol/Μl) (Macrogen, Seoul, Korea), 1 *μ*L of MgCl_2_ (25 mM) (Promega, Madison, WI, USA), 0.125 *μ*L of GoTaq Flexi DNA Polymerase (Promega, Madison, WI, USA), and as template 1 *µ*L of gDNA. PCR amplification was performed with the following conditions: initial denaturation at 95°C for 3 minutes, 35 cycles of denaturation at 95°C for 30 seconds, annealing at 55°C for 30 seconds, extension at 72°C for 30 seconds, and final extension at 72°C for 8 minutes. For all experiments, *Salmonella* spp. strains previously characterized in the Laboratory of Immunology and Molecular Biology were used as reference strains. The annealing temperature and extension time were defined based on the primer melting temperatures and the expected amplicon size (Table 1).

### 2.5. Molecular Detection of Virulence-Associated Genes and Integrons

The determination of twenty-three *Salmonella* virulence genes was performed by PCR using the conditions described previously. Primers sequences, annealing temperature, amplicon size, and corresponding references are listed in Table 2. For integron detection, gDNA from isolates was used as a template for the reaction, using gene-specific primer sets (Table 2). PCR conditions were as described above, and the annealing temperature is listed in Table 2.

### 2.6. Gel Electrophoresis and Visualization

All amplification products were revealed by gel electrophoresis in 2% agarose gel in 0.5x TBE buffer stained with HydraGreen™ (ACTGene, Piscataway, NJ, USA). 3 *μ*L PCR products were loaded in each well with 100 bp DNA Ladder (NEB, Ipswich, MA, USA) as the molecular weight marker. Electrophoresis was conducted at 100 V for 40 minutes using the PowerPac™ HC gel electrophoresis system (Bio-Rad, Hercules, CA, USA) containing 0.5x TBE buffer. The ENDURO™ GDS gel documentation system (Labnet International, Edison, NJ, USA) was utilized to visualize and document amplification products.

### 2.7. Statistical Analysis

All data were analyzed using Microsoft Excel. Prevalence as the ratio of positive animals to the total number of samples is expressed as a percentage [39].

## 3. Results

### 3.1. Distribution of Antibiotic Resistance Genes among *Salmonella* Serovars

Among the *β*-lactamase-encoding genes (*bla*_TEM_ and *bla*_CMY2_), only *bla*_CMY2_, which confers resistance to ampicillin, was detected in all the strains (100%), and *bla*_CMY2_, which confers resistance to ceftriaxone, was found at a high frequency (90%; *n* = 18) (Table 3). Among the genes specific for chloramphenicol, such as *catA* and *floR*, only *floR*, which confers resistance to florfenicol, was detected in 45% (*n* = 9) of *Salmonella* strains. Regarding the genes that encode aminoglycoside resistance, *aadA1* and *aadA2* were found to be in 75% (*n* = 15) and 15% (*n* = 3) of strains, respectively (Table 3).

Gene cassettes encoding resistance to trimethoprim, such as *dfrA1* and *dfrA12*, were present in 95% (*n* = 15) and 20% (*n* = 4) of strains, respectively. Regarding sulfonamide resistance, only sul1 and sul2 genes were detected in 50% (*n* = 10) of *Salmonella* strains. Moreover, among the quinolone and fluoroquinolone resistance genes evaluated, only *qnrD*, a plasmid-mediated quinolone resistance gene, was detected among *Salmonella* strains (20%; *n* = 4).

### 3.2. Distribution of Virulence-Associated Genes among *Salmonella* Serovars

**Figure 1:**
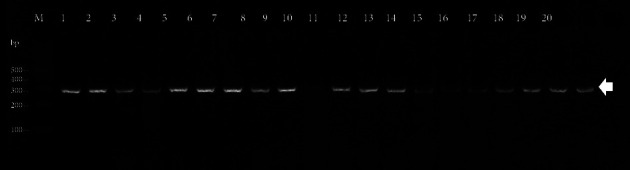
PCR amplification of a 284 bp fragment from the *invA* gene of *Salmonella* isolated from carcasses marketed in Ibague, Colombia. M: molecular-weight size marker, 100 bp DNA ladder (New England Biolabs, Ipswich, MA, USA); Lane 1: UT-SPb14007 *S*. Paratyphi B; Lane 2: UT-SPb14008 *S*. Paratyphi B; Lane 3: UT-SPb14012 *S*. Manhattan; Lane 4: UT-SPb14016 *S*. Bovismorbificans; Lane 5: UT-SPb14017 *S*. Typhimurium; Lane 6: UT-SPb14018 *S*. Typhimurium; Lane 7: UT-SPb14019 *S*. Othmarschen; Lane 8: UT-SPb14021 *S*. Newport; Lane 9: UT-SPb14024 *S*. Hvittingfoss; Lane 10: UT-SPb14027 *S*. Heidelberg; Lane 11: UT-SPb14028 *S*. Hvittingfoss; Lane 12: UT-SPb14030 *S*. Hvittingfoss; Lane 13: UT-SPb14032 *S*. Hvittingfoss; Lane 14: UT-SPb14033 *S*. Hvittingfoss; Lane 15: UT-SPb14034 *S*. Paratyphi B; Lane 16: UT-SPb14035 *S*. Muenster; Lane 17: UT-SPb14037 *S*. Muenster; Lane 18: UT-SPb14039 *S*. Muenster; Lane 19: UT-SPb14043 *S*. Paratyphi B; Lane 20: UT-SPb14047 *S*. Paratyphi B.

All 20 *Salmonella* isolates were assessed by PCR for virulence genes presence. All *Salmonella* strains amplified the expected DNA fragment of gene operon invasion A (*InvA*) that was used to confirm the *Salmonella* genus (Figure 1). In most isolates, all SPI-1 genes were present; *fimA*, *sitC*, *spaN*, *sipB*, *hilA*, and *avrA* were found to be 100%, 80% (*n* = 16), 80% (*n* = 16), 65% (*n* = 13), 100%, 85% (*n* = 17), 100%, and 90% (*n* = 18) in the isolates tested, respectively. In addition, virulence genes located on SPI-3 (*msgA*), SPI-5 (*sopB*), and SPI-7 (*sopE*) were present in all the serotypes. Likewise, virulence genes located on SPI-4, such as *siiD* (60%; *n* = 12), and SPI-6, such as *sefA* (65%; *n* = 13), were present at a lower frequency of strains.

Moreover, the frequencies of virulence genes were in non-SPI category. Genes located on the *Salmonella* virulence plasmid, such as *spvC* and *spvB*, were present in 55% (*n* = 11) of the *Salmonella* isolates. In addition, the *cdtB* gene encoding typhoid toxins was present in 95% (*n* = 19) of the isolates. Furthermore, the fimbrial genes *lpfC*, *pefA*, *lpfA*, *pagC*, and *csgA* were found in 90% (*n* = 18), 70% (*n* = 14), 90% (*n* = 18), 85% (*n* = 17), and 90% (*n* = 18) of the isolates tested, respectively.

### 3.3. Distribution of Class 1, 2, and 3 Integrons

**Figure 2:**
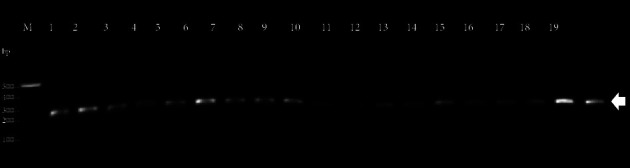
PCR amplification of a ≈247 bp fragment from the *intI2* gene of *Salmonella* isolated from carcasses marketed in Ibague, Colombia. M: molecular-weight size marker, 100 bp DNA ladder (New England Biolabs, Ipswich, MA, USA). Lane 1: UT-SPb14007; Lane 2: UT-SPb14008; Lane 3: UT-SPb14012; Lane 4: UT-SPb14016; Lane 5: UT-SPb14017; Lane 6: UT-SPb14018; Lane 7: UT-SPb14019; Lane 8: UT-SPb14021; Lane 9: UT-SPb14024; Lane 10: UT-SPb14028; Lane 11: UT-SPb14030; Lane 12: UT-SPb14032; Lane 13: UT-SPb14033; Lane 14: UT-SPb14034; Lane 15: UT-SPb14035; Lane 16: UT-SPb14037; Lane 17: UT-SPb14039; Lane 18: UT-SPb14043; Lane 19: UT-SPb14047.

Class 1 and 3 integrons were not detected in any of the *Salmonella* strains. In contrast, class 2 integrons were present in 19 strains (Figure 2).

## 4. Discussion

Poultry meat is one of the most economical and consumable sources of animal protein [40, 41]. However, poultry meat production involves several stages before the meat reaches consumers [42]. Among the stages of poultry production, food safety is an important consideration because of the bacterial pathogens present in broilers, such as *Salmonella* [43]. Contaminated poultry products are the major source of human *Salmonella* infection [44]. Contamination can occur at various stages during production on the farm (preharvest stage), during transportation to the processing plant, at slaughter and evisceration (processing stage), post-evisceration processing, cutting and boning, packaging and storage, distribution and retail, and transportation and handling [45, 46]. Also, *Salmonella* strains isolated from poultry products exhibit high levels of resistance to antibiotics, making disease control difficult and posing serious risks to global public health [47]. To minimize the impact of *Salmonella*-related outbreaks, determining the susceptibility of the bacteria to antimicrobial agents is essential [48].

Among the 20 *Salmonella* strains, antimicrobial resistance- and virulence-associated genes were determined to explore the molecular mechanisms underlying multidrug resistance (MDR) and pathogenicity. The most common resistance genes found were those encoding for *β*-lactams (*bla*_TEM_ and *bla*_CMY−2_), tetracycline (*dfrA1*), streptomycin (*aadA1*), and sulfonamide (*sul1* and *sul2*). The family of *β*-lactam antibiotics is the most prescribed family of antibiotics prescribed to treat bacterial infections [49]. Nevertheless, their use has been limited by the emergence of bacteria with resistance mechanisms, such as *β*-lactamases [50]. Some mechanisms by which bacteria acquire resistance to *β*-lactams include the production of *β*-lactamases, efflux pumps, and alterations in penicillin-binding proteins [51]. The *β*-lactamase-encoding genes *bla*_TEM_ and *bla*_CMY−2_ were predominant among the antimicrobial resistance genes detected. These findings are similar to previous studies that have reported *bla*_TEM_ and *bla*_CMY−2_ as the main genes involved in the mechanisms of resistance to *β*-lactam antibiotics and cephalosporins, respectively [52, 53]. This differs with the prevalence rates reported in a previous study conducted in China on *Salmonella* isolated from chickens, where the rates of *bla*_TEM_ and *bla*_CMY−2_*β*-lactamase genes were 61.11%, and 63.89%, respectively.

Regarding sulfamethoxazole resistance (*sul*) genes, high positivity rates for *sul1* (50%), and *sul2* (50%) were observed, which is in accordance with previous reports that indicate *sul1* and *sul2* genes are the most frequently detected *Sul* genes in *Salmonella* spp. [54, 55]. This is relevant because sulfonamide resistance usually arises from the acquisition of *sul1* and *sul2* genes, which encode forms of dihydropteroate synthase that are not inhibited by the drug [56]. Also, in Brazil, 68% of *Salmonella* isolated from chicken meat samples contained *sul2* [57]. The detection of *sul* genes in *Salmonella* strains isolated from carcasses is relevant because of the potential transfer of these genes from commensal bacteria into more virulent bacteria via integrons, transposons, or plasmids [58, 59]. Concerning chloramphenicol resistance genes, the *floR* gene was found in 45% of the *Salmonella* strains. In addition, in previous studies from China, *floR* was identified in 35.1% of *Salmonella* strains isolated from chickens, ducks, and pigs [60]. The frequency of this gene could be related to the long-term use of florfenicol in veterinary medicine, leading to the appearance of these resistance genes [61]. For example, *floR* is particularly prevalent in multidrug-resistant strains of *Salmonella* isolated from poultry [52].

Dihydrofolate reductase gene (*dfrA1*), which conferred resistance to trimethoprim (integron-encoded dihydrofolate reductase), was commonly detected between *Salmonella* strains (95%). Also, high positivity rates for *dfrA1* gene were reported on isolates recovered from clinical and environmental samples [62]. A comparative study conducted in Iraq reported 77.6% of isolates resistant to *dfrA1* gene [63]. Furthermore, 15 (75%) *Salmonella* strains contained the aminoglycoside resistance gene *aadA1*, which is relevant because of the association between *aadA1* and streptomycin resistance in *Salmonella* strains isolated from chicken meat [64]. Likewise, its frequency is also relevant because this gene is encoded by a conjugal plasmid, which can be transferred to *E. coli*, with or without selective pressure from antibiotics [65]. Regarding plasmid-mediated quinolone resistance (PMQR) genes, this study identified high frequency of *qnrD* (20%) compared to a previous report from Colombia [33]. Genetic elements, such as integrons, are able to recognize and capture cassettes carrying the antibiotic resistance genes, leading to the spread of multidrug resistance (MDR) [66]. Class 2 integrons are one of the most common integrons in pathogenic bacteria [67]. Also, the presence of integrons in *Salmonella* has been associated with antimicrobial resistance [60]. In this study, class 2 integron was detected in 95% of *Salmonella* isolates, which was higher than previous studies in South Africa (33%) and Iran (9.2%) [68, 69].

Pathogenic *Salmonella* strains isolated in poultry contain various genes associated with *Salmonella* pathogenicity islands (SPIs), including pathogenicity islands 1 and 2 (SPI-1 and SPI-2) [44]. SPI-1 allows the bacteria to penetrate non-phagocytic host cells, while SPI-2 is important for survival within the macrophage and for establishment of systemic infection [70, 71]. The SPI-1 genes, such as *fimA*, *invA*, and *avrA*, were detected in 100% of the tested strains, followed by other genes such as *spiC* (90%), *hilA* (85%), *sitC* (80%), *spaN* (80%), and *sipB* (65%), with lower frequencies (Table 4). The extensive presence of *invA* in *Salmonella* species has led to its use as a molecular marker for confirming *Salmonella* genus [72, 73]. Regarding *Salmonella* fimbriae virulence gene *fimA*, the prevalence rates were similar to a previous study in United States [74]. The *fimA* codes for the major fimbrial subunit in *Salmonella*, which helps to adhere to the cell surface and promotes colonization [75]. Another relevant gene in *Salmonella* virulence is *hilA*, which stimulates the expression of invasive genes [76]. In addition, similar results have been reported from commercial farms in Egypt, where the *hilA* gene was detected in 90% of *Salmonella* strains [77]. Likewise, the prevalence of *avrA* in strains is a concern because this gene can enhance bacteria proliferation during infections by inhibiting inflammation and regulating epithelial apoptosis [78]. Among SPI-2, various genes were detected (*sifA* (70%); *spiA* (90%)) (Table 4). The *spiA* gene is associated with the ability of biofilm formation and virulence in *Salmonella* species [79]. In this way, the presence of SPI-1 and SPI-2 genes among *Salmonella* strains isolated from poultry meat is significant, as it suggests the potential of these isolates to cause infections in humans [80].

Concerning SPI-3, *msgA* gene was present in all strains (Table 4), which agrees with previous reports on *Salmonella* isolated from poultry products in Iran [81]. By contrast, *siiD* gene of SPI-4 was found only in 60% of strains, which is relevant because this gene encodes a protein that links the inner and outer membranes [82]. Contributing to the virulence of *Salmonella* through the transport of specific proteins that enhance the bacteria's ability to invade host cells, evade the host immune system, and establish infections [83]. Another gene present among the strains was *sopB* of SPI-5, which is related to subverting host autophagy and inhibiting the fusion of *Salmonella*-containing vacuoles (SCVs) with lysosomes and autophagosomes [84]. In contrast, a study conducted in South Africa found that 31.8% of the isolates had *sopB* [85]. Therefore, the presence of *sopB* could facilitate the dissemination of bacteria in the host environment by inducing diarrhea during infection [86]. The detection rate of virulence plasmid-borne genes, such as *spvB* and *spvC*, was 55%, which was significantly higher than that in a previous report of *Salmonella* isolated from broiler chicken carcasses in the United States (5.5%) [87]. The *spvB* gene in *Salmonella* is associated with virulence and pathogenesis by promoting necroptosis of intestinal epithelial cells, leading to the destruction of the intestinal barrier and aggravation of infection [88]. The *cdtB* gene is another commonly detected gene. This gene encodes the cytolethal distending toxin B, which plays a significant role in the pathogenesis of *Salmonella* by causing cell cycle arrest, cytoplasmic distension, and nuclear enlargement in host cells [89]. Fimbriae virulence genes, such as *lpfC*, *lpfA*, and *pefA*, mediate the adhesion of *Salmonella* serovar to host cells contributing to the pathogenesis [90]. A high frequency of fimbriae virulence genes was observed among *Salmonella* strains, which is relevant because these genes contribute to inflammation, intestinal colonization, and long-term carriage of *Salmonella* in vertebrate animals [90, 91].

With respect to *csgA* gene, this gene encodes the major structural component of curli fimbriae, which stabilizes cell-cell interactions during biofilm formation [92]. *Salmonella* biofilm formation can enhance bacterial resistance to adverse conditions, such as environmental stresses, host defense mechanisms, and antibiotics, which is relevant because the detection rate of the *csgA* gene in *Salmonella* strains isolated from chicken meat was 90%, suggesting that *Salmonella* could be maintained on inert surfaces, such as those used in food production, being one of the main vehicles of foodborne salmonellosis outbreaks, which constitutes a public health problem [93]. Finally, it is essential to recognize that the presence of virulence genes is not a conclusive indicator of a bacterium's pathogenic potential [94]. It is the combined expression of multiple genes that is necessary for pathogenicity [95, 96]. Finally, we recommend using additional methods to confirm the expression of genes or proteins related to virulence factors.

## 5. Conclusions

*Salmonella* strains obtained from chicken meat containing antimicrobial and virulence genes raise concerns about their potential to cause disease and the health risk for humans. The possibility of cross-contamination in retail chicken is especially alarming, as it represents a significant danger to public health, particularly for individuals undergoing antimicrobial therapy due to *Salmonella*-contaminated chicken infections. Thus, this research holds significance for the monitoring of salmonellosis.

## Figures and Tables

**Table 1:** Primer sequences used for amplification of resistance genes.

Antimicrobial family	Antibiotic	Gene	Primer sequences (5′-3′)	Amplicon size (bp)	References
*β*-Lactams	Ceftriaxone	*bla CMY2*	F-AAATCGTTATGCTGCGCTCT	224	[32]
			R-CCGATCCTAGCTCAAACAGC		
	Ampicillin	*bla*_TEM_	F-ATCAGTTGGGTGCACGAGTG	236	
			R-ACGCTCACCGGCTCCAGA		
Chloramphenicol	Chloramphenicol	*catA*	F-CCAGACCGTTCAGCTGGATA	454	
			R-CATCAGCACCTTGTCGCCT		
	Florfenicol	*floR*	F-CACGTTGAGCCTCTATATGG	888	
			R-CACGTTGAGCCTCTATATGG		
Aminoglycoside	Streptomycin	*aadA1*	F-CTCCGCAGTGGATGGCGG	631	
			R-GATCTGCGCGCGAGGCC		
		*aadA2*	F-CATTGAGCGCCATCTGGAAT	500	
			R-ACATTTCGCTCATCGCCGGC		
Tetracycline	Trimethoprim	*dfrA1*	F-CAATGGCTGTTGGTTGGAC	254	
			R-CCGGCTCGATGTCTATTGT		
		*dfrA12*	F-TTCGCAGACTCACTGAGGG	330	
			R-CGGTTGAGACAAGCTCGAAT		
Sulfonamide	Sulfamethoxazole	*sul1*	F-CGGACGCGAGGCCTGTATC	591	
			R-GGGTGCGGACGTAGTCAGC		
		*sul2*	F-GCGCAGGCGCGTAAGCTGAT	514	
			R-CGAAGCGCAGCCGCAATTC		
		*sul3*	F-GGGAGCCGCTTCCAGTAAT	500	
			R-TCCGTGACACTGCAATCATTA		
Quinolone	Quinolone and fluoroquinolone	*qnrD*	F-CGAGATCAATTTACGGGGAATA	582	[33]
			R-AACAAGCTGAAGCGCCTG		
		*qnrS*	F-ACGACATTCGTCAACTGCAA	417	
			R-TAAATTGGCACCCTGTAGGC		
		*aac* (*6*′)-*Ib* *qnrD*	F-TTGCGATGCTCTATGAGTGGCTA	482 582	
			F-CGAGATCAATTTACGGGGAATA		

**Table 2:** Primer sequences used for amplification of virulence genes.

Category	Gene	Broad action	Primer sequences (5′-3′)	Annealing temperature (°C)	Amplicon size (bp)	References
*SPIs*						
SPI-1	*fimA*	Fimbriae	F: CCTTTCTCCATCGTCCTGAA	55	85	[34]
			R: TGGTGTTATCTGCCTGACCA			
	*sitC*	Iron metabolism	F-CAGTATATGCTCAACGCGATGTGGGTCTCC	58	768	[35]
			R-CGGGGCGAAAATAAAGGCTGTGATGAAC			
	*spaN*		F-AAAAGCCGTGGAATCCGTTAGTGAAGT	55	504	
			R-CAGCGCTGGGGATTACCGTTTTG			
	*sipB*		F-GGACGCCGCCCGGGAAAAACTCTC	58	875	
			R-ACACTCCCGTCGCCGCCTTCACAA			
	*invA*		F-GTGAAATTATCGCCACGTTCGGGCAA	55	284	
			R-TCATCGCACCGTCAAAGGAACC			
	*hilA*	Regulatory protein—(TTSS)	F-CTGCCGCAGTGTTAAGGATA	50	497	
			R-CTGTCGCCTTAATCGCATGT			
	*avrA*	Effector protein—the invasion-(TTSS)	F-AGCCTGGCGCTCGCCAAAAA	57	123	[36]
			R-GCGGTCTGCTTTATCGGACGGG			

SPI-2	*spiA*	Survival inside cells	F-CCAGGGGTCGTTAGTGTATTGCGTGAGATG	56	550	[35]
			R-CGCGTAACAAAGAACCCGTAGTGATGGATT			
	*sifA*	Effector protein—the invasion-(TTSS)	F-TTTGCCGAACGCGCCCCCACACG	58	449	
			R-GTTGCCTTTTCTTGCGCTTTCCACCCATCT			
SPI-3	*msgA*	Survival inside cells	F-GCCAGGCGCACGCGAAATCATCC	57	189	
			R-GCGACCAGCCACATATCAGCCTCTTCAAAC			

SPI-4	*siiD*	Adaptor protein—(T1SS)	F: GTCAGGGCGTTATCACTACTAAA	55	826	[37]
			R: TTCACATCGGCCAGCATAG			

SPI-5	*sopB*	Effector protein—the invasion-(TTSS)	F-CGGACCGGCCAGCAACAAAACAAGAAGAAG	55	220	[35]
			R-TAGTGATGCCCGTTATGCGTGAGTGTATT			
SPI-6	*sefA*	Fimbriae	F-GATACTGCTGAACGTAGAAGG	54	488	
			R-GCGTAAATCAGCATCTGCAGTAGC			

SPI-7	*sopE*	Effector protein—the invasion-(TTSS)	F-GAGGGCCGGGCAGTGTTGAC	55	121	[36]
			R-CTTCACGGGTCTGGCTGGCG			

*Non-SPI genes*						
Plasmid	*spvC*	PSLT plasmid	F: ACTCCTTGCACAACCAAATGCGGA	59	572	[34]
			R: TGTCTTCTGCATTTCGCCACCATCA			
	*spvB*		F-CTATCAGCCCCGCACGGAGAGCAGTTTTTA	58	717	[35]
			R-GGAGGAGGCGGTGGCGGTGGCATCATA			
Toxins	*cdtB*	Non-SPI genes	F-ACAACTGTCGCATCTCGCCCCGTCATT	57	268	
			R-CAATTTGCGTGGGTTCTGTAGGTGCGAGT			
Fimbriae	*lpfC*	Adhesins other adhesins	F-GCCCCGCCTGAAGCCTGTGTTGC	57	641	
			R-AGGTCGCCGCTGTTTGAGGTTGGATA			
	*pefA*		F-GCGCCGCTCAGCCGAACCAG	55	157	
			R-GCAGCAGAAGCCCAGGAAACAGTG			
	*lpfA*		F-CTTTCGCTGCTGAATCTGGT	57	250	
			R-CAGTGTTAACAGAAACCAGT			
	*pagC*		F-CGCCTTTTCCGTGGGGTATGC	55	454	
			R-GAAGCCGTTTATTTTTGTAGAGGAGATGTT			
	*csgA*	Adhesins curli fibers (AGF)	F-TCCACAATGGGGCGGCGGCG	54	350	
			R-CCTGACGCACCATTACGCTG			

*Integrons*						
*intI1*			F-TCCACGCATCGTCAGGC	55	280	[38]
			R-CCTCCCGCACGATGATC			

*intI2*			F-GGCAGACAGTTGCAAGACAA	57	247	[32]
			R-AAGCGATTTTCTGCGTGTTT			
*intI23*			F-CCGGTTCAGTCTTTCCTCAA	57	155	
			R-GAGGCGTGTACTTGCCTCAT			

**Table 3:** Genotypic antibiotic resistance profiles of *Salmonella* spp. isolates.^*∗*^

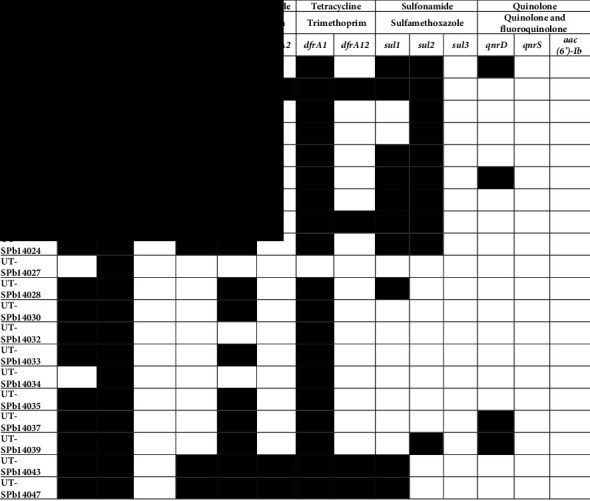

^∗^For PCR-based patterns, black box indicates that the detection of the resistance gene was positive and white box indicates that the detection of the resistance gene was negative. Abbreviations: Ceft, Ceftriaxone; Amp, Ampicillin; Chlor, Chloramphenicol; Flor, Florfenicol.

**Table 4:** Distribution of virulence genes in *Salmonella* spp. isolates.^*∗*^

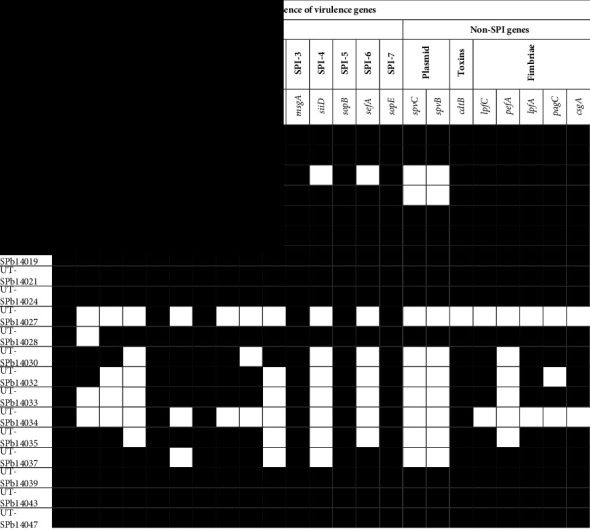

^∗^For PCR-based patterns, black box indicates that the detection of the virulence gene was positive and white box indicates that the detection of the virulence gene was negative.

## Data Availability

The data that support the findings of this study are available from the corresponding author upon reasonable request.
